# Social Category Modulation of the Happy Face Advantage

**DOI:** 10.1177/01461672241310917

**Published:** 2025-01-20

**Authors:** Douglas Martin, Ewan Bottomley, Jacqui Hutchison, Agnieszka E. Konopka, Gillian Williamson, Rachel Swainson

**Affiliations:** 1 University of Aberdeen, UK; 2 The Open University, Milton Keynes, UK

**Keywords:** social categorization, emotion categorization, social cognition, person perception, face processing, happy face advantage

## Abstract

The size of the happy face advantage—faster categorization of happy faces—is modulated by interactions between perceiver and target social categories, with reliable happy face advantages for ingroups but not necessarily outgroups. The current understanding of this phenomenon is constrained by the limited social categories typically used in experiments. To better understand the mechanism(s) underpinning social category modulation of the happy face advantage, we used racially more diverse samples of perceivers and target faces and manipulated the intergroup context in which they appeared. We found evidence of ingroup bias, with perceivers often showing a larger happy face advantage for ingroups than outgroups (Experiments 1–2). We also found evidence of majority/minority group bias, with perceivers showing a larger happy face advantage for majority outgroups than minority outgroups (Experiments 2–3c). These findings suggest social category modulation of the happy face advantage is a dynamic context-dependent process.

## Introduction

The aim of the current research is to establish whether social category modulation of the happy face advantage is a dynamic context-dependent process. The happy face advantage refers to a tendency for people to categorize happy faces more quickly or accurately than other facial expressions (Leppänen & Hietanen, 2004). Recent research found the presence and size of the happy face advantage are influenced by interactions between perceiver and target social categories, with reliable happy face advantages in reaction times for race and sex ingroup targets but not necessarily for outgroup targets (Martin et al., 2024). The conclusion of this research was that emotion categorization is a dynamic intergroup process, with the presence and size of the happy face advantage dependent on the contextually dependent relationship between the category membership of perceivers and targets. However, this theoretical interpretation is severely constrained by a lack of social diversity in the stimuli, participants, and intergroup contexts used when demonstrating these effects. Addressing these issues, the current research sought to establish whether social category modulation of the happy face advantage generalizes across different social categories and across different intergroup contexts. In so doing, the research aims to shed further light on the theoretical mechanism(s) underpinning these effects.

There is extensive evidence that the happy face advantage is affected by the apparent race and sex of a target face (for a review, see Martin et al., 2024). When target faces are White, there is near universal support for a happy face advantage, with happy White targets categorized more quickly than angry White targets (Hugenberg, 2005). When target faces are Black, the evidence is more mixed, with some studies finding a happy face advantage (Craig et al., 2012, Experiments 1d and 2b), some studies finding no such difference (Craig et al., 2012, Experiments 1c, 2a, and 3a), and some studies finding an angry face advantage whereby angry Black targets are categorized more quickly than happy Black targets (Craig et al., 2012, Experiment 1a; Hugenberg, 2005). When target faces are female, there is extensive evidence of a happy face advantage, with happy female faces categorized more quickly than angry female faces across many studies (Becker et al., 2007). When target faces are male, the evidence is more mixed, with some studies finding a happy face advantage (Craig & Lipp, 2017, Experiment 2), some studies finding no such difference (Craig & Lipp, 2017, Experiment 1), and some studies finding an angry face advantage whereby angry male faces are categorized more quickly than happy male faces (Becker et al., 2007).

While it is clear the happy face advantage is influenced by the social category of the target face, theoretical interpretation of these findings is constrained by imbalances in the race and gender of both participant samples and target faces (i.e., most participants have been White females, and most Black targets have been men; for a review, see Martin et al., 2024). Such imbalances are particularly problematic because people often perceive others differently dependent on whether they belong to the same category as themselves (i.e., the ingroup) or a different category (i.e., the outgroup; Kawakami et al., 2022; Tajfel & Turner, 1979; for a review see Dovidio & Gaertner, 2010). There is abundant evidence that people tend to respond more positively to other people when they belong to the ingroup than when they belong to an outgroup (see Dovidio & Gaertner, 2010). There is also evidence of ingroup advantages in emotion recognition, with people better at recognizing emotional expressions on target faces that belong to the same national, ethnic, or regional group to themselves (see Elfenbein & Ambady, 2002 for a meta-analysis). If ingroup bias influences emotion recognition, then experimental imbalances in the perceiver and target groups used could fundamentally influence the evidence base and associated theorizing.

Recent research found evidence to support the possibility that the happy face advantage is influenced by interactions between the social category membership of perceivers and target faces (Martin et al., 2024). Martin and colleagues recruited race- and gender-matched samples (i.e., equal numbers of Black women, Black men, White women, White men) and examined their ability to categorize happy and angry faces that were drawn from the same social categories. They found Black perceivers showed a happy face advantage for both Black and White targets, yet White perceivers showed a happy face advantage only for White targets. However, Martin et al. also found that interactions between perceiver and target categories were eliminated when faces appeared in separate single-category blocks (e.g., an entire block of Black male faces, followed by an entire block of Black female faces and so on), with perceivers from all categories showing an equivalent happy face advantage to targets from all categories (i.e., no evidence of ingroup bias). These results suggest the presence or size of the happy face advantage is not a consequence of a perceiver’s stable responses to a specific category, but is instead dependent on the social context in which faces from that category are encountered.

Clearly, the happy face advantage can be influenced by interactions between perceiver and target race, yet the generalizability and theoretical interpretation of these findings are constrained by the limited number of race categories used in demonstrating these effects (i.e., only Black & White perceivers and targets; Martin et al., 2024). For example, it is possible that White perceivers are unusual because they do not show a reliable happy face advantage for Black targets, or that Black perceivers are unusual because they show a reliable happy face advantage that is independent of target race. Similarly, it is possible that White targets are unusual because they are associated with a reliable happy face advantage that is independent of perceiver race, or that Black targets are unusual because they are only associated with a reliable happy face advantage when perceivers are Black. Thus, the perceiver-target race interactions seen in Martin et al. might reflect quirks of the specific race categories used in the experiments, rather than a dynamic intergroup process, whose effects one would expect to generalize across different social categories.

Social category modulation of the happy face advantage is often explained in terms of evaluative congruence (Craig et al., 2017; Hugenberg & Sczesny, 2006). The basic principle of the evaluative congruence explanation is that people should find it easier to categorize emotions if the valence of the emotional expression is congruent with their evaluation of the category to which a face belongs. For example, the larger happy face advantages often found for female and White targets have been attributed to perceivers’ relatively more positive evaluations of women relative to men (Eagly et al., 1991) and perceivers “own race” relative to “other races” (it is worth noting that in previous research, “own race” and “other races” have been predicated on most participants being White; Degner & Wentura, 2010; see Lindeberg et al., 2019). While evaluative congruence is a plausible explanation for the cognitive mechanism underpinning experimental demonstrations of social category modulation of emotion categorization, it seems underspecified as a social cognitive mechanism. Indeed, there are many social cognitive phenomena that might result in evaluative congruence. For example, evaluative congruence might be driven by top-down factors, such as relatively stable individual differences in a person’s stereotype knowledge or attitudes (Bijlstra et al., 2010), bottom-up factors, such as those associated with the perceptual features of a face (Becker et al., 2007), or by more transient contextually dependent factors, such as those associated with intergroup bias (Dovidio & Gaertner, 2010). Any of these factors, and others besides, might have the ability to drive evaluative congruence, with social context determining which dominates in a given setting (Freeman & Ambady, 2011).

## Current Research

The overarching aim of the current research is to establish whether social category modulation of the happy face advantage is a dynamic intergroup process. Across five experiments, we asked participants to make speeded emotion categorizations of happy and angry target faces (Target Emotion), with the faces being categorized comprising equal numbers of unfamiliar faces from two social category dimensions (Target Race and Target Sex). We manipulated the intergroup relationship between the race of perceivers and targets across the experiments (i.e., we varied whether targets were ingroup race/sex or outgroup race/sex relative to perceivers). The aim of Experiments 1 and 2 was to establish whether ingroup bias might lead to social category modulation of the happy face advantage that generalizes across different race categories; in these experiments, half of the target faces belonged to a race ingroup and half of the faces belonged to a race outgroup relative to perceivers (i.e., Experiment 1: Chinese and White perceivers categorizing Chinese and White targets; Experiment 2: Black and Chinese perceivers categorizing Black and Chinese targets). The aim of Experiments 3a to 3c was to establish whether social category modulation of the happy face advantage might be driven by factors relating to the influence of majority/minority cultural exposure; in these experiments, all the faces belonged to race outgroups relative to perceivers (i.e., Experiment 3a: Chinese perceivers categorizing Black and White targets; Experiment 3b: Black perceivers categorizing Chinese and White targets; Experiment 3c: White perceivers categorizing Black and Chinese targets); NB: our participants were all located in the United Kingdom, which meant they were embedded within a White majority cultural environment (i.e., ~83% White, ~4% Black, ~1%, Chinese; Office for National Statistics [ONS], 2022).

## Experiment 1

The aim of Experiment 1 was to establish whether social category modulation of the happy face advantage generalizes across different race categories. Recent research suggests people show reliable happy face advantages for ingroups but not necessarily outgroups (Martin et al., 2024). However, because these effects were only demonstrated using two race categories (i.e., Black and White), it is not clear whether they generalize to other race categories. If social category modulation of the happy face advantage is a dynamic context-dependent process—and, therefore, dependent on perceiver/target category overlap—we would expect interactions between perceiver race and target race and evidence of ingroup bias. We would also expect to see analogous interactions between perceiver sex and target sex (Martin et al., 2024).

### Method

This research design and analysis strategy were not preregistered. We report all manipulations, measures, and exclusions in these studies. Participant instructions, the data, analysis code, and a codebook for all experiments can be found here: https://osf.io/8ewxd/.

#### Design

Experiment 1 had a 2 (Perceiver Race: Chinese perceivers vs. White perceivers) × 2 (Perceiver Sex: female perceivers vs. male perceivers) × 2 (Target Race: Chinese targets vs. White targets) × 2 (Target Sex: female targets vs. male targets) × 2 (Target Emotion: angry targets vs. happy targets) mixed factorial design, with Perceiver Sex and Perceiver Race as between-subjects factors. We used the data from Experiment 1 by Martin et al. (2024) and the package mixedpower (Kumle et al., 2021) to provide parameters to estimate the minimum sample size required to detect the predicted Perceiver Category × Target Category × Target Emotion interactions. The simulation showed that a sample size of 200 provided >83% power for the detection of a three-way interaction between Perceived Sex, Target Race, and Target Emotion. To provide a fully counterbalanced experimental design of the experiment with ~80% power, we aimed to have a final sample of at least 192 participants.

#### Participants

Data from 197 participants were included in the final sample (48 Chinese women, 48 Chinese men, 48 White women, and 53 White men; age range = 18–40; age *M* = 25 years). We aimed to have a final sample of around 192 participants, with approximately equal numbers of Chinese women, Chinese men, White women, and White men. In previous research, ~8% of participants were excluded from the original samples because they exhibited excessively high error rates or excessively slow reaction times (both >3 *SD* above the median; Martin et al., 2024). To ensure we had a gender and race-balanced sample, we over-recruited our initial sample by around 10% to allow us to exclude outlier participants based on their performance relative to the entire sample, while still leaving sufficient power to test our hypotheses. To this end, we initially recruited 212 young adult participants (53 from each perceiver category), via the online recruitment platform Prolific Academic (www.prolific.ac). Participants completed the experiment remotely via the online testing platform Gorilla (www.gorilla.sc) and were compensated around UK£2.50 for their time. We used prescreen criteria available in Prolific Academic to recruit only participants who self-identified as “Located in the UK,” their *Sex* as “Female” or “Male,” and their *Ethnicity* and *Nationality* as “East Asian/South-East Asian” and “Born in China” or “White/Caucasian” and “Born in the United Kingdom.” We excluded participants if they exhibited excessively high error rates or excessively slow reaction times (both >3 *SD* above the median); this resulted in the exclusion of 15 participants.

#### Materials and Procedure

Participants were informed at the recruitment stage that they would be taking part in a study examining the speed and accuracy with which people can categorize emotional faces. Participants were tested online and we had no control over the physical environment in which they were tested.

The target faces comprised 256 color digital headshot images of 128 unfamiliar people; 64 Chinese targets were selected from the Tsinghua Facial Expression Database (Yang et al., 2020), and 64 White targets were selected from the Chicago Face Database (Ma et al., 2015). Faces were chosen by the lead researcher on the subjective basis that they were unambiguously representative of each social category (pilot data indicated people were able to rapidly identify the correct sex and race of each target). The face images included hair. The overall image was cropped to a standardized size of 200 × 240 pixels (on a 1,280 × 1,024 screen resolution); the Gorilla testing platform standardized the size ratio of targets across different screen sizes by asking participants to calibrate their screen size using a credit card); participants completed the experiment on a laptop or desktop PC. Target faces were drawn equally from four social categories: Chinese females, Chinese males, White females, and White males. There were two images of each target face, one with a happy expression (i.e., smiling) and one with an angry expression (i.e., frowning). This meant there were 32 images from eight distinct social category and emotion subtypes—happy Chinese females, angry Chinese females, happy Chinese males, angry Chinese males, happy White females, angry White females, happy White males, and angry White males. Participants saw each identity only once, with either a happy or angry expression.

The experiment consisted of a single block of 128 trials. Each trial comprised the presentation of a fixation cross for 500 ms followed by a target face image for 300 ms after which the image disappeared. The intertrial interval was 1,200 ms. Participants made their responses using keyboard keys (“V” and “B”). The meaning of the response buttons was counterbalanced across participants. The order of trial presentation was randomized, and the computer recorded the latency and accuracy of responses.

#### Dependent Measures and Analysis Strategy

We used *R* (R Core Team, 2021) and *lme4* (Bates et al., 2015) to perform linear mixed effects analyses of the relationship between correct emotion categorization Reaction Times (RTs)^1^ and perceiver and target factors. Raw correct RTs were analyzed using linear mixed effects regression (lmer), with mean RTs described for the analyses and reported in both Tables and Figures.

### Results

There were a total of 25,216 valid trials across the 197 participants. Reaction times ranged from 11 ms to 54 s. We excluded 33 trials where reaction times were likely to be too fast to reflect a deliberative response to the stimulus (i.e., < 250 ms; see Martin et al., 2024); error rate on these trials was around chance (*M* errors = .48). We also excluded 175 trials where reaction times were likely to be too slow to reflect attendance to the task (i.e., >1,500 ms; Martin et al., 2024); the error rate on these trials was high (*M* errors = .24). We visually inspected the distribution of correct RTs (*M* = 519 ms; standard deviation [*SD*] = 157 ms), which revealed a long-right-hand tail of relatively longer reaction times; we addressed this issue by excluding trials with response times greater than 3 *SD*s away from the sample mean, which resulted in the exclusion of a further 457 response times greater than 1,067 ms. Participants accurately categorized emotional expression on 94% of remaining trials; we excluded from the reaction time analysis trials on which errors were made (1,482 trials excluded). Thus, the final data sets comprised 24,551 responses for the error analysis (see Supplemental Error Analyses) and 23,069 correct response times for the RT analysis (see below).

We used *R* v.4.3.2 (R Core Team, 2021) and *lme4* v.1.1.35.1 (Bates et al., 2015) to perform linear mixed effects analyses of the relationship between correct emotion categorization reaction times and perceiver and target factors. The model with the maximal random structure justified by the design included a five-way interaction between the five categorical variables of interest as well as random by-participant and by-item intercepts, random by-participant slopes for Target Sex, Target Race, and Target Emotion, and random by-item slopes for Perceiver Sex (Brauer & Curtin, 2018). Regression-style contrast coding (−.5 and .5 for the two levels of each variable, with reference levels determined alphabetically) was used for all categorical variables. The bound optimization by quadratic approximation (bobyqa) with a set maximum of 200,000 iterations was used. When singularity issues were encountered, the random effect structure was simplified (by iteratively removing random slopes for the variable that accounted for the smallest amount of variance until convergence was reached). Including slopes for all variables resulted in singular fit warnings, with the exception of by-participant slopes for Target Emotion. The most complex model to converge included the specified fixed effects plus random by-participant slopes for Target Emotion and no by-item random slopes:

$$lmer\left(\begin{array}{l} RT\sim 1+PerceiverRace*PerceiverSex\\ {}*TargetRace*TargetSex*TargetEmotion\\ +\left(1+TargetEmotion|participant\right)+\left(1|item\right) \end{array}\right)$$

We report the model with the highest-level interactions between fixed effects and a reduced random effects structure that only includes random slopes improving model fit (i.e., Target Emotion). Full output for this model can be found in Supplemental Table 1a. The model indicated some significant main effects and interactions that were subsumed by higher order interactions or that were not predicted or of central theoretical interest. However, we restrict our description of the model to the predicted interactions between Target/Perceiver Categories and Target Emotion.

### Interaction of Perceiver Race, Target Race, and Target Emotion

**Figure 1.**
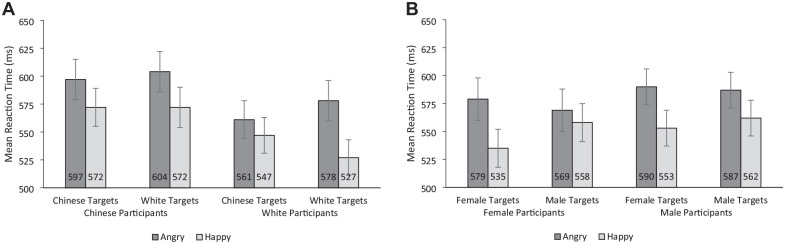
Experiment 1 Mean Reaction Times (A) by Perceiver Race, Target Race, and Target Emotion and (B) by Perceiver Sex, Target Sex, and Target Emotion. *Note.* Error bars represent 95% confidence intervals.

The RT model indicated the presence of the predicted Perceiver Race × Target Race × Target Emotion interaction (*b* = 29, *t* = 4.98, *p* < .001; see Figure 1A). We further examined this interaction by running separate Target Race × Target Emotion tests for each Perceiver Race. For Chinese perceivers, there was no evidence of a Target Race × Target Emotion interaction (*b* = 8, *t* = 1.02, *p* = .31) but there was evidence of an overall happy face advantage, as indicated by a significant main effect of Target Emotion (*b* = 29, *t* = 5.58, *p* < .001). In contrast, for White perceivers there was evidence of a Target Race × Target Emotion interaction (*b* = 37, *t* = 5.27, *p* < .001), with a significantly larger happy face advantage for White targets (*M* diff = 51 ms, [95% confidence interval [CI] = 37, 64]) than Chinese targets (*M* diff = 14 ms, [95% CI = 0, 27]).

### Interaction of Perceiver Sex, Target Sex, and Target Emotion

The RT model indicated the presence of the predicted Perceiver Sex × Target Sex × Target Emotion interaction (*b* = 20, *t* = 3.49, *p* < .001; see Figure 1B). We further examined this interaction by running separate Target Sex × Target Emotion models for each Perceiver Sex. For female perceivers, there was evidence of a Target Sex × Target Emotion interaction (*b* = 33, *t* = 4.52, *p* < .001), with a significantly larger happy face advantage for female targets (*M* diff = 45 ms, [95% CI = 30, 59]) than male targets (*M* diff = 11 ms, [95% CI = −3, 26]). In contrast, for male perceivers, there was no evidence of a Target Sex × Target Emotion interaction (*b* = 13, *t* = 1.77, *p* = .079) but there was evidence of an overall happy face advantage, as indicated by a significant main effect of Target Emotion (*b* = 33, *t* = 6.85, *p* < .001).

### Discussion

The aim of Experiment 1 was to establish whether social category modulation of the happy face advantage generalizes across different race categories. As hypothesized, we found evidence that the happy face advantage was influenced by interactions between perceiver race and target race for Chinese/White perceivers and targets. Chinese perceivers showed no difference in the size of the happy face advantage for Chinese and White targets, whereas White perceivers showed evidence of ingroup bias, with a significantly larger happy face advantage for White targets than Chinese targets. These findings conceptually replicate and extend previous findings for Black and White perceivers and targets (Martin et al., 2024). As predicted, we also replicated previous evidence of interaction between perceiver sex and target sex (Martin et al., 2024). Male perceivers showed an overall happy face advantage for both female and male targets, whereas female perceivers showed evidence of ingroup bias, with a significantly larger happy face advantage for female targets than male targets.

The results from Experiment 1 support the idea that social category modulation of the happy face advantage is a dynamic context-dependent process and not specific to Black and White perceivers and targets. The overall pattern of effects is strikingly similar to previous findings (Martin et al., 2024). Chinese perceivers in the current research and Black perceivers in Martin et al. showed no difference in the size of the happy face advantage for ingroup race targets and outgroup White targets, whereas White perceivers in the current research and in Martin et al. showed a significantly larger happy face advantage for ingroup race targets than outgroup targets (either Chinese or Black). That White perceivers show a larger happy face advantage for ingroup race targets than outgroup race targets supports an ingroup bias explanation. However, Black and Chinese perceivers do not show a larger happy face advantage for ingroup race targets than outgroup race targets does not seem to support an ingroup bias explanation. It is possible that the mixed pattern of intergroup bias found across different perceiver groups is because of the additional influence of majority/minority group status; we explore this in Experiment 2.

## Experiment 2

The aim of Experiment 2 was to establish whether, in the absence of a majority outgroup, ingroup bias might drive social category modulation of the happy face advantage. There is evidence that people’s ingroup bias is dependent on whether the ingroup and outgroup are a majority or minority group (Griffiths & Nesdale, 2006; Pektas et al., 2023). Griffiths and Nesdale found that people from a majority ethnic group evaluated their ingroup more positively than two ethnic minority outgroups (i.e., ingroup bias). In contrast, people from the minority groups were equally positive in their ratings of their ingroup and the ethnic majority outgroup (i.e., no ingroup bias), but evaluated their ingroup more positively than an ethnic minority outgroup (i.e., ingroup bias). If one extends the logic from evaluative ratings to the happy face advantage, one might expect ethnic minority perceivers to show a larger happy face advantage for an ingroup minority than an outgroup minority (i.e., ingroup bias). To establish this, in Experiment 2 we investigated whether Black and Chinese perceivers would show evidence of ingroup race bias when the experimental context did not contain ethnic majority White targets (i.e., only Black and Chinese perceivers and targets). If there is evidence of ingroup bias, we hypothesized we would see a Perceiver Race × Target Race × Target Emotion interaction, with both Black and Chinese perceivers showing a larger happy face advantage for ingroup race targets than outgroup race targets.

### Method

The method used in Experiment 2 was identical to that used in Experiment 1, with two exceptions based on manipulation of Perceiver Race and Target Race: (a) The race of all perceivers was either Black, or ethnic Chinese, and (b) the race of all targets was either Black or ethnic Chinese.

#### Participants

Data from 204 participants were included in the final sample (53 Black women, 48 Black men, 52 Chinese women, and 51 Chinese men; age range = 18–40; age *M* = 26 years). We initially recruited 212 young adult participants (53 from each perceiver category), via the online recruitment platform Prolific Academic (www.prolific.ac). Participants completed the experiment remotely via the online testing platform Gorilla (www.gorilla.sc) and were compensated around UK£2.50 for their time. We used prescreen criteria available in Prolific Academic to recruit only participants who self-identified as “Located in the UK,” self-identified their *Sex* as “Female” or “Male,” and their *Ethnicity* and *Nationality* as or “Black/African American/Black British” and “Born in the USA/Born in the United Kingdom” or “East Asian/South-East Asian” and “Born in China.” We excluded participants if they exhibited excessively high error rates or excessively slow reaction times (both >3 *SD* above the median); this resulted in the exclusion of eight participants.

#### Materials and Procedure

Participants were tested individually online with an identical procedure to Experiment 1. The only difference in the materials was that the White target face stimuli from Experiment 1 were replaced with Black target faces (the Black faces were previously used by Martin et al., 2024).

### Results

There were a total of 26,112 valid trials across the 204 participants. Reaction times ranged from 3 ms to 61 s. We removed 747 outlying trials (see Experiment 1 for method). Participants accurately categorized emotional expression on 95% of remaining trials; for the reaction time analysis, we excluded trials on which errors were made (1,400 trials excluded). Thus, the final data sets comprised 25,365 for responses for the error analysis (see Supplemental Error Analyses) and 23,965 correct response times for the RT analysis (see below). To ensure consistency across experiments, we report the data from the model that included the specified fixed effects plus random by-participant slopes for Target Emotion and no by-item random slopes. The full output for both above model can be found in Supplemental Table 2a.

### Interaction of Perceiver Race, Target Race, and Target Emotion

**Figure 2.**
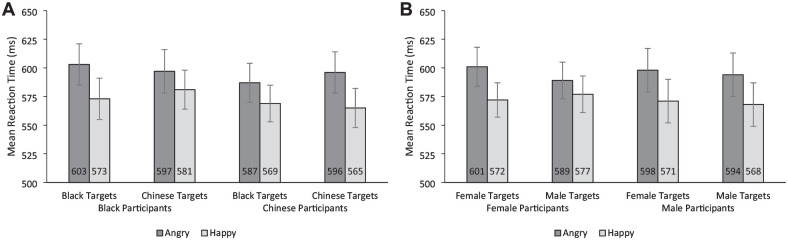
Experiment 2 Mean Reaction Time (A) by Participant Race, Target Race, and Target Emotion and (B) by Perceiver Sex, Target Sex, and Target Emotion. *Note.* Error bars represent 95% confidence intervals.

The RT model indicated evidence of the predicted Perceiver Race × Target Race × Target Emotion interaction (*b* = 28, *t* = 4.90, *p* < .001; see Figure 2A). We further examined this interaction by running separate Target Race × Target Emotion models for each Perceiver Race. For Black perceivers, there was evidence of a Target Race × Target Emotion interaction (*b* = 15, *t* = 2.58, *p* = .03), with a significantly larger happy face advantage for Black targets (*M* diff = 30 ms, [95% CI = 19, 42]) than Chinese targets (*M* diff = 16 ms, [95% CI = 4, 28]). This pattern was reversed for Chinese perceivers, who also showed evidence of a Target Race × Target Emotion interaction (*b* = 13, *t* = 2.04, *p* = .044), but showed a significantly larger happy face advantage for Chinese targets (*M* diff = 31 ms, [95% CI = 19, 43]) than Black targets (*M* diff = 18 ms, [95% CI = 6, 29]).

### Interaction of Perceiver Sex, Target Sex, and Target Emotion

The RT model indicated the presence of the predicted Perceiver Sex × Target Sex × Target Emotion interaction (*b* = 14, *t* = 2.39, *p* = .017; see Figure 2B). We further examined this interaction by running separate Target Sex × Target Emotion models for each Perceiver sex. For female perceivers, there was evidence of a Target Sex × Target Emotion interaction (*b* = 16, *t* = 2.38, *p* = .019), with a significantly larger happy face advantage for female targets (*M* diff = 28 ms, [95% CI = 17, 40]) than male targets (*M* diff = 13 ms, [95% CI = 1, 24]). In contrast, for male perceivers, there was no evidence of a Target Sex × Target Emotion interaction (*b* = 2, *t* = .24, *p* = .81) but there was evidence of an overall happy face advantage, as indicated by a significant main effect of Target Emotion (*b* = 27, *t* = 6.27, *p* < .001).

### Discussion

The aim of Experiment 2 was to establish whether, in the absence of a majority outgroup, ingroup bias might drive social category modulation of the happy face advantage. Mirroring previous findings of ingroup bias in explicit evaluations of minority ingroups and outgroups (Griffiths & Nesdale, 2006; Pektas et al., 2023), we found evidence of larger happy face advantages for minority ingroup targets than minority outgroup targets. As hypothesized, we found significant a Perceiver Race × Target Race × Target Emotion interactions. Both Chinese and Black participants had a significantly larger happy face advantage for their race ingroup, compared with their outgroup. As with Experiment 1, we also replicated the Perceiver Sex, Target Sex, and Target Emotion interaction, with a similar happy face advantage for both male and female faces when the perceiver is male, but a larger happy face advantage for female faces when the perceiver is female (Martin et al., 2024). When the results from Experiment 2 are considered in the context of those from Experiment 1 and previous findings (Martin et al., 2024), these findings suggest social category modulation of the happy face advantage is a dynamic intergroup process.

In the absence a majority race outgroup, as was the case in Experiment 2, the pattern of social category modulation of the happy face advantage follows the pattern one would expect from ingroup bias—a larger happy face advantage for race ingroup targets. In contrast, in the presence of a majority race outgroup, as was the case in Experiment 1 and in previous research (e.g., Experiments 2a and 2b by Martin et al., 2024), the results followed the pattern of majority/minority group influence—no larger happy face advantage for race ingroup targets. Given that removing majority race targets from the experimental context enabled us to observe the influence of ingroup bias in social category modulation of emotion categorization, it is possible that removing ingroup bias from the experimental context might enable us to observe the influence of majority/minority influence. We explore this possibility in Experiments 3a to 3c.

## Experiments 3a to 3c

The aim of Experiments 3a to 3c was to determine whether, in the absence of ingroup bias, majority/minority influence might drive social category modulation of the happy face advantage. A feature of Experiments 1 and 2, and of most other demonstrations of the perceiver by target race interaction (Craig & Lipp, 2017; Martin et al., 2024), is that ingroup race is *always* present within the experimental context. However, in the absence of the ingroup from the experimental context, if people evaluate majority outgroups more positively than minority outgroups (Griffiths & Nesdale, 2006), one might expect to see a larger happy face advantage for the majority group. Experiments 3a to 3c examine whether perceivers show interactions between Target Race and Target Emotion when ingroup race is not present in the experimental context. Specifically, they investigate the emotion categorization of outgroup faces for a sample of Chinese (3a), Black (3b), and White (3c) participants. Given previous evidence that people report more positive evaluative ratings for majority outgroups than minority outgroups (Griffiths & Nesdale, 2006), we hypothesized Chinese perceivers would show a larger happy face advantage for race majority White targets than race minority Black targets (Experiment 3a), and that Black perceivers would show a larger happy face advantage for race majority White targets than race minority Chinese targets (Experiment 3b). As White perceivers were faced with an experimental context where targets were both drawn from outgroups, so where neither intergroup bias or majority/minority influence are applicable, we did not anticipate an interaction between Target Race and Target Emotion (Experiment 3c). As in Experiments 1 and 2, we also expected to see interactions between perceiver sex and target sex (Martin et al., 2024).

### Method—Experiment 3a

The method used in Experiment 3a was identical to that used in previous research (Experiments 2a and 2b from Martin et al., 2024), with the exception that all perceivers self-identified their race/ethnicity as “Chinese.”

#### Participants

The data from 95 participants were included in the final sample (47 ethnic Chinese females and 48 ethnic Chinese males; age range = 18–39; age *M* = 26 years). We initially recruited 106 young adult participants, via the online recruitment platform *Prolific Academic* (www.prolific.ac). Participants completed the experiment remotely via the online testing platform *Gorilla* (www.gorilla.sc) and were compensated around UK£2.50 for their time. We used prescreen criteria available in Prolific Academic to recruit only participants who self-identified as “Located in the UK,” their *Sex* as “Female” or “Male,” and their *Ethnicity* and *Nationality* as “East Asian/South-East Asian” and “Born in China.” We excluded participants if they exhibited excessively high error rates or excessively slow reaction times (both >3 *SD* above the median); this resulted in the exclusion of 11 participants.

#### Design

The experiment had a 2 (Perceiver Sex: female perceivers vs. male perceivers) × 2 (Target Race: Black targets vs. White targets) × 2 (Target Sex: female targets vs. male targets) × 2 (Target Emotion: angry targets vs. happy targets) mixed factorial design, with Perceiver Sex as a between-subjects factor.

#### Materials and Procedure

The general procedure was identical to Experiments 1 and 2, with identical trial and block procedures. The only difference from Experiments 1 and 2 was in the composition of the target stimuli, which comprised the White target images from Experiment 1 and the Black target images from Experiment 2. The materials and procedure were identical to those used in previous research (Experiments 2a and 2b from Martin et al., 2024), but crucially, perceivers were sampled from a different ethnic background (i.e., Chinese perceivers).

### Results—Experiment 3a

There were a total of 12,160 valid trials across the 95 participants. Reaction times ranged from 5 ms to 27 s. We initially removed 321 outlying trials (see Experiment 1 for method). Participants accurately categorized emotional expression on 95% of remaining trials; we excluded from the RT analysis trials on which errors were made (584 trials excluded). Thus, the final data sets comprised 11,839 responses for error analysis (see Supplemental Error Analyses) and 11,255 correct response times for RT analysis (see below). To ensure consistency across experiments, we report the data from RT model that included the specified fixed effects plus random by-participant slopes for Target Emotion and no by-item random slopes; the model converged. The full output for both above model can be found in Supplemental Table 3a.

### Interaction of Target Race and Target Emotion

**Figure 3.**
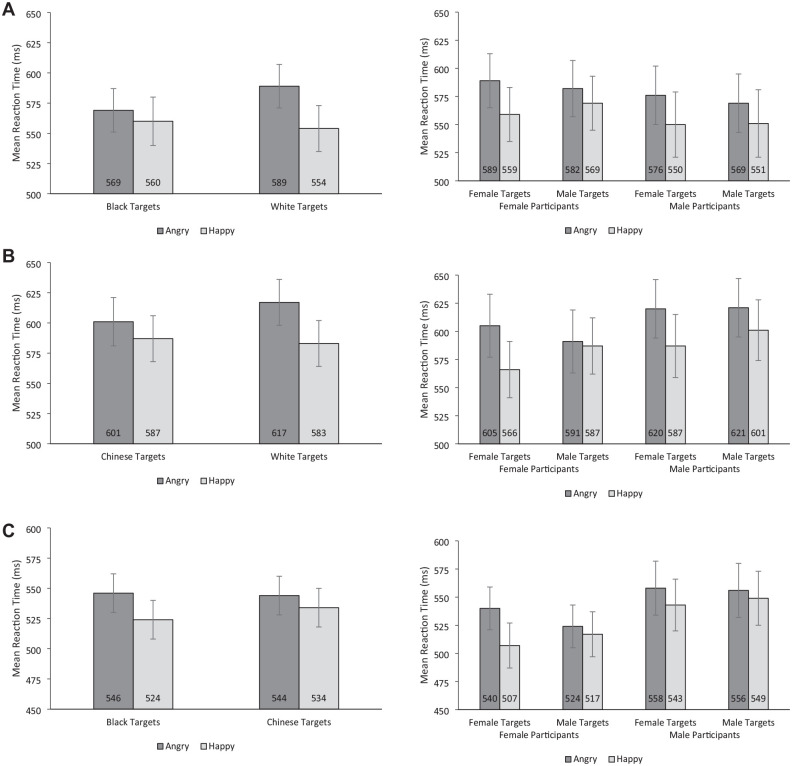
Mean Reaction Times for Chinese Perceivers (Ai) by Target Race and Target Emotion and (Aii) by Perceiver Sex, Target Sex, and Target Emotion; Mean Reaction Times for Black Perceivers (Bi) by Target Race and Target Emotion and (Bii) by Perceiver Sex, Target Sex, and Target Emotion; Mean Reaction Times for White Perceivers (Ci) by Target Race and Target Emotion and (Cii) by Perceiver Sex, Target Sex, and Target Emotion. *Note.* Error bars represent 95% confidence intervals.

The RT model indicated the presence of a Target Race × Target Emotion interaction (*b* = 26, *t* = 3.34, *p* = .001; see Figure 3Ai), with a significantly larger happy face advantage for White targets (*M* diff = 35, [95% CI = 23, 48]) than Black targets (*M* diff = 9, [95% CI = −4, 21]).

### Interaction of Perceiver Sex, Target Sex, and Target Emotion

The RT model indicated there was no evidence of the predicted Perceiver Sex × Target Sex × Target Emotion interaction (*b* = 10, *t* = 1.30, *p* = .195; see Figure 3Aii), nor was there any evidence of an interaction of Target Sex × Target Emotion (*b* = 12.36, *t* = 1.56, *p* = .121). However, there was evidence of an overall happy face advantage, as indicated by a significant main effect of Target Emotion (*b* = 22, *t* = 4.57, *p* < .001).

### Method—Experiment 3b

The method used in Experiment 3b was identical to that used in Experiment 1, with the exception that all perceivers self-identified their race/ethnicity as “Black.”

#### Participants

Data from 103 participants were included in the final sample (52 Black females and 51 Black males; age range = 18–30; age *M* = 24 years). We initially recruited 106 UK young adult participants, via the online recruitment platform *Prolific Academic* (www.prolific.ac). Participants completed the experiment remotely via the online testing platform *Gorilla* (www.gorilla.sc) and were compensated around UK£2.50 for their time. We used prescreen criteria available in Prolific Academic to recruit only participants who self-identified as “Located in the UK,” self-identified their *Sex* as “Female” or “Male,” and their *Ethnicity* and *Nationality* as or “Black/African American/Black British” and “Born in the USA/Born in the United Kingdom.” We excluded participants if they exhibited excessively high error rates or excessively slow reaction times (both >3 *SD* above the median); this resulted in the exclusion of three participants.

#### Design

The experiment had a 2 (Perceiver Sex: female perceivers vs. male perceivers) × 2 (Target Race: Chinese targets vs. White targets) × 2 (Target Sex: female targets vs. male targets) × 2 (Target Emotion: angry targets vs. happy targets) mixed factorial design, with Perceiver Sex as a between-subjects factor.

#### Materials and Procedure

The materials and procedure were identical to those used in Experiment 1.

### Results—Experiment 3b

There were a total of 13,184 valid trials across the 103 participants. Reaction times ranged from 2 ms to 203 s. We initially removed 484 outlying trials (see Experiment 1 for method). Participants accurately categorized emotional expression on 95% of remaining trials; we excluded from the RT analysis trials on which errors were made (670 trials excluded). Thus, the final data sets comprised 12,700 responses for the error analysis (see Supplemental Error Analyses) and 12,030 correct response times for the RT analysis (see below). To ensure consistency across experiments, we report the data from the model that included the specified fixed effects plus random by-participant slopes for Target Emotion and no by-item random slopes; the model converged. The full output for the model can be found in Supplemental Table 3bi.

### Interaction of Target Race, and Target Emotion

The RT model indicated the presence of a Target Race × Target Emotion interaction (*b* = 21, *t* = 2.60, *p* = .011; see Figure 3Bi, top panel), with a significantly larger happy face advantage for White targets (*M* diff = 34, [95% CI = 22, 47]) than Chinese targets (*M* diff = 14, [95% CI = 1, 26]).

### Interaction of Perceiver Sex, Target Sex, and Target Emotion

The RT model indicated the presence of the predicted Perceiver Sex × Target Sex × Target Emotion interaction (*b* = 21, *t* = 2.37, *p* = .018; see Figure 3Bii). We further examined this interaction by running separate Target Sex × Target Emotion models for each Perceiver Sex. For female perceivers, there was evidence of a Target Sex × Target Emotion interaction (*b* = 35, *t* = 3.73, *p* < .001), with a significantly larger happy face advantage for female targets (*M* diff = 39 ms, [95% CI = 22, 56]) than male targets (*M* diff = 4 ms, [95% CI = −14, 21]. In contrast, for male perceivers, there was no evidence of a Target Sex × Target Emotion interaction (*b* = 14, *t* = 1.46, *p* = .15) but there was evidence of an overall happy face advantage, as indicated by a significant main effect of Target Emotion (*b* = 27, *t* = 4.31, *p* < .001).

### Method—Experiment 3c

The method used in Experiment 3c was identical to that used in Experiment 2, with the exception that all perceivers self-identified their race/ethnicity as “White.”

#### Participants

Data from 103 participants were included in the final sample (50 White females and 53 White males; age range = 17–34; age *M* = 22 years). We initially recruited 106 young UK young adult participants, via the online recruitment platform *Prolific Academic* (www.prolific.ac). Participants completed the experiment remotely via the online testing platform *Gorilla* (www.gorilla.sc) and were compensated around UK£2.50 for their time. We used prescreen criteria available in *Prolific Academic* to recruit only participants who self-identified as “Located in the UK,” “Female” or “Male,” and “White/Caucasian.” We excluded participants if they exhibited excessively high error rates or excessively slow reaction times (both >3 *SD* above the median); this resulted in the exclusion of three participants.

#### Design

The experiment had a 2 (Perceiver Sex: female perceivers vs. male perceivers) × 2 (Target Race: Black targets vs. Chinese targets) × 2 (Target Sex: female targets vs. male targets) × 2 (Target Emotion: angry targets vs. happy targets) mixed factorial design, with Perceiver Sex as a between-subjects factor.

#### Materials and Procedure

The materials and procedure were identical to those used in Experiment 2.

### Results—Experiment 3c

There were a total of 13,184 valid trials across the 103 participants. Reaction times ranged from 52ms to 35 s. We initially removed 285 outlying trials (see Experiment 1 method). Participants accurately categorized emotional expression on 94% of remaining trials; we excluded from the RT analysis trials on which errors were made (786 trials excluded). Thus, the final data sets comprised 12,899 responses for the error analysis (see Supplemental Error Analyses) and 12,133 correct response times for the RT analysis (see below). To ensure consistency across experiments, we report the data from the model that included the specified fixed effects plus random by-participant slopes for Target Emotion and no by-item random slopes; both the RT and errors models converged. The full output for both above model can be found in Supplemental Table 3ci.

### Interaction of Target Race and Target Emotion

The RT model indicated the presence of a Target Race × Target Emotion interaction (*b* = 12, *t* = 2.15, *p* = .034; see Figure 3Ci), with a significantly larger happy face advantage for Black targets (*M* Diff = 22, [95% CI = 13, 31]) than Chinese targets (*M* Diff = 10, [95% CI = 1, 19]).

### Interaction of Perceiver Sex, Target Sex, and Target Emotion

The RT model indicated the presence of the predicted Perceiver Sex × Target Sex × Target Emotion interaction (*b* = 19, *t* = 2.63, *p* < .001; see Figure 3Cii). We further examined this interaction by running separate Target Sex × Target Emotion models for each Perceiver sex. For female perceivers, there was evidence of a Target Sex × Target Emotion interaction (*b* = 26, *t* = 3.89, *p* < .001), with a significantly larger happy face advantage for female targets (*M* diff = 34 ms, [95% CI = 21, 47]) than male targets (*M* diff = 7 ms, [95% CI = −.6, 20]). In contrast, for male perceivers, there was no evidence of a Target Sex × Target Emotion interaction (*b* = 7, *t* = 1.05, *p* = .30) but there was evidence of an overall happy face advantage, as indicated by a significant main effect of Target Emotion (*b* = 11, *t* = 2.61, *p* = .011).

### Discussion—Experiments 3a to 3c

The aim of Experiments 3a to 3c was to determine whether, in the absence of intergroup bias, majority/minority influence might drive social category modulation of the happy face advantage. We found evidence that this was the case, with interactions between Target Race and Target Emotion in all three experiments. As hypothesized, in the absence of ingroup Chinese targets (Experiment 3a), Chinese perceivers showed a significantly larger happy face advantage for majority outgroup White targets than minority outgroup Black targets. Similarly, in the absence of ingroup Black targets (Experiment 3b), Black participants showed a significantly larger happy face advantage for majority outgroup White targets than minority outgroup Chinese targets. In the absence of ingroup White targets (Experiment 3c), White perceivers showed a significantly larger happy face advantage for minority outgroup Black targets than minority outgroup Chinese targets. We once again replicated the perceiver sex, target sex, and target emotion interaction; in two of the three experiments, male perceivers showed a similar happy face advantage for both male and female faces, whereas female perceivers showed a happy face advantage only for female faces (Martin et al., 2024).

## General Discussion

The overarching aim of the current research was to establish whether social category modulation of the happy face advantage is a dynamic context-dependent process—we found evidence to support this idea. Extending previous findings (Martin et al., 2024), we found evidence that social category modulation of the happy face advantage generalizes across different race categories (Experiments 1 and 2) and different intergroup contexts (Experiments 3a–3c). We also provide novel theoretical insight into the potential mechanisms underlying these effects, with evidence to support both ingroup bias and majority/minority group influence as drivers of race modulation of emotion categorization. It is also worth noting that across four of five experiments, we replicated previous evidence of interactions between Perceiver Sex, Target Sex, and Target Emotion (Martin et al., 2024).

The current findings provide support for the role of ingroup bias as a potential contributory factor in driving the effects of social category modulation of the happy face advantage (Martin et al., 2024). Across each of the five experiments, perceivers showed a happy face advantage for targets that were ingroup race or ingroup sex. Supporting an ingroup favoritism explanation, there were larger happy face advantages for ingroup than outgroup race targets for White perceivers (Experiment 1), Black perceivers (Experiment 2), and Chinese perceivers (Experiment 2); the only exception to this was Chinese perceivers who showed no difference in the size of the happy face advantage for ingroup Chinese targets and outgroup White targets (Experiment 1). The ingroup favoritism explanation fits with the evaluative congruence account of social category modulation of emotion categorization (Craig et al., 2017; Hugenberg, 2005; Hugenberg & Sczesny, 2006), whereby people often evaluate the ingroup more favorably than the outgroup (both at the level of individual people and the categories themselves; for a review, see Dovidio & Gaertner, 2010). If people hold a relatively more positive evaluation of ingroups, one might expect them also to show a larger happy face advantage for targets from ingroup categories.

While ingroup bias can explain many of the findings of the current and previous research, it does not account for all the findings. Replicating and extending previous research, all perceiver groups showed a large and reliable happy face advantage toward White targets irrespective of whether they were an ingroup or outgroup (for a review, see Martin et al., 2024). For Chinese perceivers, the happy face advantage for outgroup White targets was equivalent to that for ingroup race targets (Experiment 1) and was larger than that for outgroup Black targets (Experiment 3a). Similarly, for Black perceivers, the happy face advantage for outgroup White targets was larger than that for outgroup Chinese targets (Experiment 3b). This pattern of results mirrors findings from research examining evaluative ratings of majority/minority groups (Griffiths & Nesdale, 2006; Pektas et al., 2023), whereby members of ethnic minority groups evaluate a White-majority outgroup as positively as their ethnic ingroup and more positively than another minority ethnic outgroup. It is also analogous to previous research in face recognition, which found people from minority race groups were not only significantly more accurate at recognizing ingroup faces than outgroup faces, they also showed a consistent trend to be more accurate at recognizing faces from outgroup majority groups than outgroup minority groups (Vingilis-Jaremko et al., 2020). While majority/minority influence might explain the current pattern of results for race, further research is required to elucidate the real-world social factors that underpin this effect; for example, these effects might be underpinned by relative differences in the perceived societal status of majority/minority groups (Griffiths & Nesdale, 2006) or relative differences in statistical learning through enculturation (Turk-Browne, 2012; explored below).

The current research also supports and extends previous evidence that emotion categorization is influenced by interactions between the sex of perceivers and target faces (Martin et al., 2024). In four of our five experiments, we found evidence that female perceivers show a larger happy face advantage for ingroup female targets than male targets, whereas male perceivers show no difference in the size of the happy face advantage for female and male targets. Importantly, by increasing the race diversity of both perceivers and target faces, and the experimental context in which they appear, the current research demonstrates that these interactions are generalizable and not tied to White perceivers and targets. Larger happy face advantages for female faces than male faces are often attributed to evaluative congruence (Hugenberg & Sczesny, 2006), which is driven by more positive societal evaluations of women (Eagly et al., 1991). However, this explanation alone is insufficient to explain why male perceivers fail to show a larger happy face advantage for female targets than male targets. Instead, it seems like there might be multiple drivers of these effects, with these drivers exerting divergent influence across different contexts.

While research to date supports the idea that social category modulation of the happy face advantage is driven by multiple social cognitive drivers, we remain open to the possibility that all these drivers might stem from a single overarching mechanism. One potential candidate for such a mechanism might be statistical learning from enculturation (Turk-Browne, 2012). The population of the United Kingdom, from where our participant samples were drawn, is predominantly ethnically White (~83% White; ONS, 2022), with much smaller proportions of people who identify as ethnically Black (~4%) or ethnically Chinese (~1%); thus, if our perceivers encounter an unfamiliar person in their daily lives, that person is most likely to be White. There is also evidence to suggest women smile more often than men in both laboratory-based studies (LaFrance et al., 2003) and in everyday life (Chapell, 1997; McDuff et al., 2017). Smiling White women are also used more often than other social categories in news articles (Kwak & An, 2016) and advertising campaigns (An & Kwak, 2019). Therefore, if one lives in the United Kingdom and encounters an unfamiliar smiling face, statistically that face is more likely to belong to a White female than any other race and gender combination. Seeing a relatively larger number of unfamiliar smiling White women in daily life might lead to perceptual and cognitive efficiencies for categorizing such faces, with White women becoming the social category default association of a smiling face. However, it is also possible seeing a relatively larger number of unfamiliar smiling White women might lead to the development of more positive evaluations of the categories White and female. Any of these perceptual, cognitive, or evaluative explanations might explain the substantial happy face advantages for White and female targets that we find irrespective of intergroup status. Exposure to more unfamiliar smiling white faces in daily life might also explain why Black and Chinese perceivers show a larger happy face advantage for outgroup White targets than targets from another outgroup race (i.e., Experiments 3a and 3b). Similarly, exposure to a relatively larger number of unfamiliar smiling Black faces in daily life might explain why, in the in the absence of White targets, White perceivers showed a larger happy face advantage for outgroup Black targets than outgroup Chinese targets (i.e., Experiment 3c).

While the current research provides novel theoretical insight and widens the evidence base to increase generalizability, it also has several limitations which still constrain the generalizability of the findings. First, the presented research did not control for aspects of the target stimuli that may vary across target categories, such as perceived age, attractiveness, and prototypicality, each of which may impact the social category modulation of emotion categorization. Second, our experiment only included happy and angry faces. As different emotions can elicit different biases (Bijlstra et al., 2010), it may be the case that alternative results would be obtained when examining faces with other emotional expressions. Third, the perceivers and targets in the current research, who were all aged under thirty, who identified with a single binary biological sex, and who identified as belonging to a discrete race group rather than being of mixed race, still represent a relatively narrow subsection of the wider population. While the same diversity limitations can be said to be true of the samples used in most psychological research, they would seem particularly important in studies of social categories. Fourth, the scope of the current research was restricted to examining the effects of two social category dimensions independently (i.e., race and sex) and not the intersection between these or other categories. Addressing intersectionality in appropriately powered future research seems particularly pertinent given previous evidence that emotional expressions influence gender and race categorization of intersectional targets (Smith et al., 2017). As the evidence base grows, future research should strive to address the above limitations and ensure theoretical interpretation of findings are appropriately constrained.

## Conclusion

The current findings suggest that social category modulation of the happy face advantage is a dynamic context-dependent process. While evaluative congruence remains a plausible explanation for experimental differences in the size of the happy face advantage, it seems unlikely to be driven by relatively fixed evaluations. Instead, it seems more likely that evaluative congruence can be driven by a multitude of different social cognitive factors. Here, we provide evidence to support the role of both ingroup bias and majority/minority influence as potential drivers of evaluative congruence, but different contexts are likely to be sensitive to different drivers. It is also possible that there is a single overarching mechanism, such as statistical learning from enculturation, driving all these effects. When considered in the context of other research in this area, the current findings add weight to the argument that social categorization (including categorization of emotions) is not simply a task of identifying the perceptual features in a face; it is instead a process of person construal that requires the dynamic integration of bottom-up perceptual cues and top-down social information (Freeman & Ambady, 2011).

## Supplemental Material

sj-docx-1-psp-10.1177_01461672241310917 – Supplemental material for Social Category Modulation of the Happy Face Advantage Supplemental material, sj-docx-1-psp-10.1177_01461672241310917 for Social Category Modulation of the Happy Face Advantage by Douglas Martin, Ewan Bottomley, Jacqui Hutchison, Agnieszka E. Konopka, Gillian Williamson and Rachel Swainson in Personality and Social Psychology Bulletin

sj-docx-2-psp-10.1177_01461672241310917 – Supplemental material for Social Category Modulation of the Happy Face Advantage Supplemental material, sj-docx-2-psp-10.1177_01461672241310917 for Social Category Modulation of the Happy Face Advantage by Douglas Martin, Ewan Bottomley, Jacqui Hutchison, Agnieszka E. Konopka, Gillian Williamson and Rachel Swainson in Personality and Social Psychology Bulletin

sj-docx-3-psp-10.1177_01461672241310917 – Supplemental material for Social Category Modulation of the Happy Face Advantage Supplemental material, sj-docx-3-psp-10.1177_01461672241310917 for Social Category Modulation of the Happy Face Advantage by Douglas Martin, Ewan Bottomley, Jacqui Hutchison, Agnieszka E. Konopka, Gillian Williamson and Rachel Swainson in Personality and Social Psychology Bulletin

## Notes

1. For economy of space, error models are reported in the online materials (Supplemental Error Analyses). The overall pattern of interactions for errors was very similar to those reaction times across the experiments.
