# Supplementary material for: Social Category Modulation of the Happy Face Advantage
Source: Pers Soc Psychol Bull. 2025 Jan 20;52(5):1327–40. doi: 10.1177/01461672241310917 (PMC13022007; doi:10.1177/01461672241310917)
Supplement: sj-docx-1-psp-10.1177_01461672241310917 – Supplemental material for Social Category Modulation of the Happy Face Advantage [file sj-docx-1-psp-10.1177_01461672241310917.docx]

**Full experiment onscreen instructions**

This is an example from one of the counterbalanced version of the experiment (Experiment 1).

# In this experiment you will be asked to categorize faces.

# You will see images of unfamiliar faces and your task will be to categorize their emotional expressions as quickly and as accurately as you can. We would like you to categorize the faces as being either angry or happy.

# You will complete one practice block (12 trials) and one experimental block (128 trials).

# The whole experiment takes around 15 minutes to complete.

# You should use your LEFT AND RIGHT INDEX FINGERS to make your responses.

# You should have the forefinger from your left hand on V.

# You should have the forefinger from your right hand on B.

# Your task will be to categorize faces by emotion.

# If the person is **HAPPY** press **V**

# If the person is **ANGRY** press **B**

# **V = HAPPY**

# **B = ANGRY**

# You should respond as **QUICKLY** and as **ACCURATELY** as you can.

# Click below to do some practice trials.

# That's the end of the practice trials.

# Remember:

# **V = Happy**

# **B = Angry**

# Press space to begin the experimental block.
