# Supplementary material for: Social Category Modulation of the Happy Face Advantage
Source: Pers Soc Psychol Bull. 2025 Jan 20;52(5):1327–40. doi: 10.1177/01461672241310917 (PMC13022007; doi:10.1177/01461672241310917)
Supplement: sj-docx-2-psp-10.1177_01461672241310917 – Supplemental material for Social Category Modulation of the Happy Face Advantage [file sj-docx-2-psp-10.1177_01461672241310917.docx]

**Supplementary Error Analyses**

**Error analyses – Experiment 1**

We used R (R Core Team, 2021) and lme4 (Bates et al., 2012) to perform linear mixed effects analyses of the relationship between errors and perceiver and target factors. Error rates were analyzed with binomial general linear mixed effects regression (glmer), with Odds Ratios (OR) described for the analyses and reported in Tables and proportion errors reported in Supplementary Figures. The most complex model to converge for Errors included the specified fixed effects plus random by-participant slopes for Target Emotion and no by-item random slopes:

glmer (Errors ~ 1 + Perceiver Race * Perceiver Sex * Target Race * Target Sex * Target Emotion + (1 + Target Emotion |participant) + (1|item))

The full output for the above model can be found in Supplementary Table1b. The model indicated some significant main effects and interactions that were either subsumed by higher order interactions, or that were not predicted or of central theoretical interest. However, we restrict our description of the model to the predicted effects of primary theoretical interest (i.e., interactions between Target/Perceiver Categories and Target Emotion).

**Interaction of Perceiver Race, Target Race, and Target Emotion**

The Errors model indicated a trend towards the predicted Perceiver Race X Target Race X Target Emotion interaction (*b* = .43,***t* =** 1.88, *p* = .060; see Supplementary Figure 1a).

Supplementary Figure 1a. Experiment 1 mean errors by Perceiver Race, Target Race, and Target Emotion. Error bars represent 95% confidence intervals.

**Interaction of Perceiver Sex, Target Sex, and Target Emotion**

The Errors model indicated the presence of the predicted Perceiver Sex X Target Sex X Target Emotion interaction (*b* = .61,***t* =** 2.65, *p* = .008; see Supplementary Figure 1b). We further examined this interaction by running separate Target Sex X Target Emotion models for each Perceiver sex. For female perceivers, there was evidence of a Target Sex X Target Emotion interaction (*b* = .89**, *t* =** 4.42, *p* < .001), with a significantly larger happy face advantage for female (Odds Ratio = 1.84, [95% CI = 1.26, 2.69]) targets than male targets (OR = .76, [95% CI = .52, 1.09]). In contrast, for male perceivers there was no evidence of a Target Sex X Target Emotion interaction (*b* = .40**, *t* =** 1.71, *p* = .086) but there was evidence of an overall happy face advantage, as indicated by a significant main effect of Target Emotion (*b* = .40**, *t* =** 2.73, *p* = .006).

Supplementary Figure 1b. Experiment 1 mean proportion errors by Perceiver sex, Target Sex, and Target Emotion. Error bars represent 95% confidence intervals.

**Error analyses – Experiment 2**

The full output for the Errors model can be found in Supplementary Table 2b.

**Interaction of Target Race and Target Emotion**

The Errors model indicated evidence of the predicted Perceiver Race X Target Race X Target Emotion interaction (*b* = .77,***t* =** 3.44, *p* = .001; see Supplementary Figure 2a). We examined this interaction by running separate Target Race X Target Emotion tests for each Perceiver Race. For Black perceivers, there was no evidence of a significant Target Race X Target Emotion interaction (*b* = .15**, *t* =** .80, *p* = .43) or a significant main effect of Target Emotion (*b* = .20**, *t* =** 1.52, *p* = .13). For Chinese perceivers, there was evidence of a Target Race X Target Emotion interaction (*b* = .55**, *t* =** 2.62, *p* = .009), with a significantly larger happy face advantage for Chinese targets (OR = 1.44, [95% CI = .99, 2.08]) than Black targets (OR = .83, [95% CI = .58, 1.21]), *t* = 1.11, *p* = .53).

Supplementary Figure 2a. Experiment 2 Mean proportion errors by Participant Race, Target Race, and Target Emotion. Error bars represent 95% confidence intervals.

**Interaction of Perceiver Sex, Target Sex, and Target Emotion**

The Errors model also indicated the presence of the predicted Perceiver Sex X Target Sex X Target Emotion interaction (*b* = .83, ***t***= 3.70, *p* < .001; see Supplementary Figure 2b). We further examined this interaction by running separate Target Sex X Target Emotion models for each Perceiver sex. For female perceivers, there was evidence of a Target Sex X Target Emotion interaction (*b* = .89**, *t* =** 4.56, *p* < .001), with a significantly larger happy face advantage for female targets (OR = 1.60, [95% CI = 1.13, 2.27]) than male targets (OR = .66, [95% CI = .46, .94]). In contrast, for male perceivers there was no evidence of a Target Sex X Target Emotion interaction (*b* = .13**, *t* =** .62, *p* = .54), and only a trend towards a significant main effect of Target Emotion (*b* = .24**, *t* =** 1.74, *p* = .081).

Supplementary Figure 2b. Experiment 2 Mean proportion errors by Perceiver sex, Target Sex, and Target Emotion. Error bars represent 95% confidence intervals.

**Error analyses – Experiment 3a**

The full output for the errors model can be found in Supplementary Table 3aii.

**Interaction of Target Race, and Target Emotion**

The Errors model indicated the presence of a Target Race X Target Emotion interaction (*b* = .74**, *t*** = 3.02, *p* = .003; see Supplementary Figure 3ai), with a significantly larger happy face advantage for White targets (OR = 1.60, [95% CI = 1.09, 2.33]) than Black targets (OR = .76, [95% CI = .51, 1.12]).

Supplementary Figure 3ai. Experiment 3a Mean proportion errors for Chinese perceivers by Target Race and Target Emotion. Error bars represent 95% confidence intervals.

**Interaction of Perceiver Sex, Target Sex, and Target Emotion**

The Errors model indicated the presence of the predicted Perceiver Sex X Target Sex X Target Emotion interaction (*b* = .72**, *t*** = 2.06, *p* = .040; see Supplementary Figure 3aii). We further examined this interaction by running separate Target Sex X Target Emotion models for each Perceiver sex. For female perceivers, there was evidence of a Target Sex X Target Emotion interaction (*b* = .70**, *t* =** 2.22, *p* = .026), with a significantly larger happy face advantage for female targets (OR = 1.71, [95% CI = .95, 3.10]) than male targets (OR = .85, [95% CI = .47, 1.51]). In contrast, for male perceivers there was no evidence of a Target Sex X Target Emotion interaction (*b* = .08**, *t* =** .26, *p* = .80), nor was there was evidence of an overall happy face advantage, with no significant main effect of Target Emotion (*b* = .09**, *t* =** .47, *p* = .64).

Supplementary Figure 3aii. Experiment 3a Mean proportion errors for Chinese perceivers by Perceiver Sex, Target Sex and Target Emotion. Error bars represent 95% confidence intervals.

**Error analyses – Experiment 3b**

The full output for the errors model can be found in Supplementary Table 3bi Supplementary Table 3bii.

**Interaction of Target Race, and Target Emotion**

The Errors model indicated there was a Target Race X Target Emotion interaction (*b* = .48**, *t*** = 2.02, *p* = .043; see Supplementary Figure 3bi), with a significantly larger happy face advantage for White targets (OR = 1.73, [95% CI = 1.19, 2.51]) than Chinese targets (OR = 1.00, [95% CI = .69, 1.46]).

Supplementary Figure 3bi. Experiment 3b Mean proportion errors for Black perceivers by Target Race and Target Emotion. Error bars represent 95% confidence intervals.

**Interaction of Perceiver Sex, Target Sex, and Target Emotion**

The Errors model also indicated the presence of the predicted Perceiver Sex X Target Sex X Target Emotion interaction (*b* = 1.28**, *t*** = 3.92, *p* < .001; see Supplementary Figure 3bii). We further examined this interaction by running separate Target Sex X Target Emotion models for each Perceiver sex. For female perceivers, there was evidence of a Target Sex X Target Emotion interaction (*b* = .95**, *t* =** 3.06, *p* = .002), with a significantly larger happy face advantage for female targets (OR = 1.92, [95% CI = 1.09, 3.38]) than male targets (OR = .75, [95% CI = .43, 1.29]). In contrast, for male perceivers there was no evidence of a Target Sex X Target Emotion interaction (*b* = .37**, *t* =** 1.22, *p* = .22) but there was evidence of an overall happy face advantage, as indicated by a significant main effect of Target Emotion (*b* = .45**, *t* =** 2.19, *p* = .029).

Supplementary Figure 3bii. Experiment 3b Mean proportion errors (bottom panel) for Black perceivers by Perceiver Sex, Target Sex and Target Emotion. Error bars represent 95% confidence intervals.

**Results – Experiment 3c**

The full output for the errors model can be found in Supplementary Table 3cii.

**Interaction of Target Race and Target Emotion**

The Errors model indicated there was no significant Target Race X Target Emotion interaction (*b* = .08**, *t*** = .42, *p* = .68; see Supplementary Figure 3ci).

Supplementary Figure 3ci. Experiment 3c Mean proportion errors for White perceivers by Target Race and Target Emotion. Error bars represent 95% confidence intervals.

**Interaction of Perceiver Sex, Target Sex, and Target Emotion**

The Errors model also indicated the presence of the predicted Perceiver Sex X Target Sex X Target Emotion interaction (*b* = .97**, *t*** = 3.26, *p* = .001; see Supplementary Figure 3cii). We further examined this interaction by running separate Target Sex X Target Emotion models for each Perceiver sex. For female perceivers, there was evidence of a Target Sex X Target Emotion interaction (*b* = .91**, *t* =** 3.74, *p* < .001), with a significantly larger happy face advantage for female targets (OR = 1.45, [95% CI = .96, 2.20]) than male targets (OR = .58, [95% CI = .39, .88]). In contrast, for male perceivers there was no evidence of a Target Sex X Target Emotion interaction (*b* = .02**, *t* =** .06, *p* = .95) nor was there any evidence of an overall happy face advantage, with no main effect of Target Emotion (*b* = .08**, *t* =** .49, *p* = .63).

Supplementary Figure 3cii. Experiment 3c Mean proportion errors for White perceivers by Perceiver Sex, Target Sex and Target Emotion. Error bars represent 95% confidence intervals.
