# Supplementary material for: Social Category Modulation of the Happy Face Advantage
Source: Pers Soc Psychol Bull. 2025 Jan 20;52(5):1327–40. doi: 10.1177/01461672241310917 (PMC13022007; doi:10.1177/01461672241310917)
Supplement: sj-docx-3-psp-10.1177_01461672241310917 – Supplemental material for Social Category Modulation of the Happy Face Advantage [file sj-docx-3-psp-10.1177_01461672241310917.docx]

Supplementary Table 1a. Expt. 1 output for the converged RT model:

lmer (RT ~ 1 + Perceiver Sex * Perceiver Race * Target Sex * Target Race * Target Emotion + (1 + Target Emotion |Participant) + (1|item))

|  | Effect | Variance | Estimate | *SE* | *df* | *t* | *p* |
| --- | --- | --- | --- | --- | --- | --- | --- |
| Fixed Effects | |  |  |  |  |  |  |
|  | (Intercept) |  | 569.49 | 5.75 | 218 | 99.08 | <.001*** |
|  | PSex |  | 18.79 | 11.12 | 193 | 1.69 | .093 |
|  | PRace |  | -33.70 | 11.12 | 193 | -3.03 | .003** |
|  | TSex |  | 5.08 | 3.24 | 118 | 1.57 | .119 |
|  | TRace |  | 0.85 | 3.24 | 118 | 0.26 | .793 |
|  | TEmotion |  | -30.46 | 4.04 | 211 | -7.54 | <.001*** |
|  | PSex*PRace |  | 21.70 | 22.24 | 193 | 0.98 | .330 |
|  | PSex*TSex |  | -2.98 | 2.86 | 22540 | -1.04 | .297 |
|  | PRace*TSex |  | -2.12 | 2.86 | 22541 | -0.74 | .458 |
|  | PSex*TRace |  | -2.55 | 2.86 | 22540 | -0.89 | .373 |
|  | PRace*TRace |  | -5.30 | 2.86 | 22541 | -1.85 | .064 |
|  | TSex*TRace |  | 4.42 | 6.48 | 118 | 0.68 | .496 |
|  | PSex*TEmotion |  | -5.32 | 5.62 | 188 | -0.95 | .345 |
|  | PRace*TEmotion |  | -3.61 | 5.62 | 188 | -0.64 | .521 |
|  | TSex*TEmotion |  | 23.47 | 6.48 | 118 | 3.62 | <.001*** |
|  | TRace*TEmotion |  | -22.38 | 6.48 | 118 | -3.46 | <.001*** |
|  | PSex*PRace*TSex |  | 5.25 | 5.72 | 22541 | 0.92 | .358 |
|  | PSex*PRace*TRace |  | 1.66 | 5.72 | 22541 | 0.29 | .772 |
|  | PSex*TSex*TRace |  | 9.18 | 5.72 | 22535 | 1.61 | .109 |
|  | PRace*TSex*TRace |  | 1.73 | 5.72 | 22535 | 0.30 | .763 |
|  | PSex*PRace*TEmotion |  | 2.84 | 11.24 | 188 | 0.25 | .801 |
|  | PSex*TSex*TEmotion |  | -19.95 | 5.72 | 22538 | -3.49 | <.001*** |
|  | PRace*TSex*TEmotion |  | -3.60 | 5.72 | 22538 | -0.63 | .530 |
|  | PSex*TRace*TEmotion |  | -5.14 | 5.72 | 22537 | -0.90 | .369 |
|  | PRace*TRace*TEmotion |  | -28.51 | 5.72 | 22538 | -4.98 | <.001*** |
|  | TSex*TRace*TEmotion |  | 7.54 | 12.95 | 118 | 0.58 | .562 |
|  | PSex*PRace*TSex*TRace |  | -1.55 | 11.44 | 22535 | -0.14 | .892 |
|  | PSex*PRace*TSex*TEmotion |  | -21.35 | 11.44 | 22538 | -1.87 | .062 |
|  | PSex*PRace*TRace*TEmotion |  | -11.62 | 11.44 | 22538 | -1.02 | .310 |
|  | PSex*TSex*TRace*TEmotion |  | 22.53 | 11.44 | 22536 | 1.97 | .049* |
|  | PRace*TSex*TRace*TEmotion |  | -4.64 | 11.44 | 22537 | -0.41 | .685 |
|  | PSex*PRace*TSex*TRace*TEmotion |  | -3.68 | 22.88 | 22537 | -0.16 | .872 |
| Random Effects | |  |  |  |  |  |  |
|  | Item (Intercept) | 270 |  |  |  |  |  |
|  | Subject (Intercept) | 5975 |  |  |  |  |  |
|  | TEmotion | 1144 |  |  |  |  |  |
|  | Residual | 12068 |  |  |  |  |  |

Supplementary Table 1b. Expt. 1 output for the converged Error model:

glmer (Errors ~ 1 + Perceiver Sex * Target Sex * Target Race * Target Emotion + (1 + Target Emotion |Participant) + (1|item))

|  | Effect | Variance | Estimate | *SE* | *t* | *p* |
| --- | --- | --- | --- | --- | --- | --- |
| Fixed Effects | |  |  |  |  |  |
|  | (Intercept) |  | -3.16 | 0.07 | -42.61 | <.001*** |
|  | PSex |  | -0.24 | 0.13 | -1.84 | .065 |
|  | PRace |  | 0.27 | 0.13 | 2.04 | .042* |
|  | TSex |  | 0.04 | 0.08 | 0.42 | .677 |
|  | TRace |  | 0.21 | 0.08 | 2.49 | .013* |
|  | TEmotion |  | -0.23 | 0.10 | -2.20 | .028* |
|  | PSex*PRace |  | -0.40 | 0.26 | -1.53 | .127 |
|  | PSex*TSex |  | -0.06 | 0.12 | -0.53 | .594 |
|  | PRace*TSex |  | 0.15 | 0.12 | 1.26 | .206 |
|  | PSex*TRace |  | -0.04 | 0.12 | -0.32 | .750 |
|  | PRace*TRace |  | 0.02 | 0.12 | 0.21 | .835 |
|  | TSex*TRace |  | 0.16 | 0.17 | 0.95 | .340 |
|  | PSex*TEmotion |  | -0.18 | 0.15 | -1.14 | .254 |
|  | PRace*TEmotion |  | -0.04 | 0.15 | -0.29 | .772 |
|  | TSex*TEmotion |  | 0.68 | 0.17 | 4.04 | <.001*** |
|  | TRace*TEmotion |  | -0.75 | 0.17 | -4.48 | <.001*** |
|  | PSex*PRace*TSex |  | 0.01 | 0.23 | 0.06 | .956 |
|  | PSex*PRace*TRace |  | 0.34 | 0.23 | 1.49 | .137 |
|  | PSex*TSex*TRace |  | -0.04 | 0.23 | -0.18 | .856 |
|  | PRace*TSex*TRace |  | -0.28 | 0.23 | -1.21 | .225 |
|  | PSex*PRace*TEmotion |  | 0.55 | 0.31 | 1.80 | .072 |
|  | PSex*TSex*TEmotion |  | -0.61 | 0.23 | -2.65 | .008** |
|  | PRace*TSex*TEmotion |  | -0.34 | 0.23 | -1.49 | .137 |
|  | PSex*TRace*TEmotion |  | -0.23 | 0.23 | -1.01 | .312 |
|  | PRace*TRace*TEmotion |  | -0.43 | 0.23 | -1.88 | .060 |
|  | TSex*TRace*TEmotion |  | -0.52 | 0.34 | -1.55 | .121 |
|  | PSex*PRace*TSex*TRace |  | -0.96 | 0.46 | -2.08 | .038* |
|  | PSex*PRace*TSex*TEmotion |  | 0.69 | 0.46 | 1.49 | .137 |
|  | PSex*PRace*TRace*TEmotion |  | -0.70 | 0.46 | -1.51 | .131 |
|  | PSex*TSex*TRace*TEmotion |  | -0.46 | 0.46 | -1.00 | .318 |
|  | PRace*TSex*TRace*TEmotion |  | 0.70 | 0.46 | 1.52 | .129 |
|  | PSex*PRace*TSex*TRace*TEmotion |  | 0.12 | 0.92 | 0.13 | .899 |
| Random Effects | |  |  |  |  |  |
|  | Item (Intercept) | 0.12 |  |  |  |  |
|  | Subject (Intercept) | 0.64 |  |  |  |  |
|  | TEmotion | 0.41 |  |  |  |  |

Supplementary Table 2a. Expt. 2 output for the converged RT model:

lmer (RT ~ 1 + Perceiver Sex * Perceiver Race * Target Sex * Target Race * Target Emotion + (1 + Target Emotion |Participant) + (1|item))

|  | Effect | Variance | Estimate | *SE* | *df* | *t* | *p* |
| --- | --- | --- | --- | --- | --- | --- | --- |
| Fixed Effects | |  |  |  |  |  |  |
|  | (Intercept) |  | 583.69 | 5.94 | 217 | 98.27 | <.001*** |
|  | PSex |  | -1.98 | 11.61 | 200 | -0.17 | .865 |
|  | PRace |  | -8.85 | 11.61 | 200 | -0.76 | .447 |
|  | TSex |  | -3.50 | 2.88 | 117 | -1.22 | .226 |
|  | TRace |  | 1.45 | 2.88 | 117 | 0.51 | .614 |
|  | TEmotion |  | -23.63 | 3.40 | 184 | -6.95 | <.001*** |
|  | PSex*PRace |  | 15.40 | 23.23 | 200 | 0.66 | .508 |
|  | PSex*TSex |  | -0.07 | 2.87 | 23433 | -0.02 | .981 |
|  | PRace*TSex |  | 2.93 | 2.87 | 23434 | 1.02 | .307 |
|  | PSex*TRace |  | -2.12 | 2.87 | 23431 | -0.74 | .460 |
|  | PRace*TRace |  | 1.40 | 2.87 | 23431 | 0.49 | .625 |
|  | TSex*TRace |  | 6.76 | 5.75 | 117 | 1.18 | .242 |
|  | PSex*TEmotion |  | -6.26 | 4.63 | 200 | -1.35 | .178 |
|  | PRace*TEmotion |  | -1.29 | 4.63 | 200 | -0.28 | .780 |
|  | TSex*TEmotion |  | 8.56 | 5.75 | 117 | 1.49 | .140 |
|  | TRace*TEmotion |  | 1.00 | 5.75 | 117 | 0.17 | .863 |
|  | PSex*PRace*TSex |  | -6.60 | 5.74 | 23434 | -1.15 | .250 |
|  | PSex*PRace*TRace |  | 0.62 | 5.74 | 23431 | 0.11 | .914 |
|  | PSex*TSex*TRace |  | 5.99 | 5.73 | 23427 | 1.05 | .296 |
|  | PRace*TSex*TRace |  | 2.42 | 5.73 | 23428 | 0.42 | .673 |
|  | PSex*PRace*TEmotion |  | -22.38 | 9.26 | 200 | -2.42 | .017* |
|  | PSex*TSex*TEmotion |  | -13.70 | 5.74 | 23428 | -2.39 | .017* |
|  | PRace*TSex*TEmotion |  | 0.66 | 5.74 | 23428 | 0.12 | .908 |
|  | PSex*TRace*TEmotion |  | 6.36 | 5.74 | 23429 | 1.11 | .267 |
|  | PRace*TRace*TEmotion |  | -28.12 | 5.74 | 23429 | -4.90 | <.001*** |
|  | TSex*TRace*TEmotion |  | 13.34 | 11.51 | 117 | 1.16 | .249 |
|  | PSex*PRace*TSex*TRace |  | 11.44 | 11.47 | 23428 | 1.00 | .319 |
|  | PSex*PRace*TSex*TEmotion |  | 0.07 | 11.47 | 23428 | 0.01 | .995 |
|  | PSex*PRace*TRace*TEmotion |  | -1.08 | 11.47 | 23429 | -0.09 | .925 |
|  | PSex*TSex*TRace*TEmotion |  | 3.64 | 11.47 | 23427 | 0.32 | .751 |
|  | PRace*TSex*TRace*TEmotion |  | 19.71 | 11.47 | 23427 | 1.72 | .086 |
|  | PSex*PRace*TSex*TRace*TEmotion |  | -36.97 | 22.94 | 23427 | -1.61 | .107 |
| Random Effects | |  |  |  |  |  |  |
|  | Item (Intercept) | 199 |  |  |  |  |  |
|  | Subject (Intercept) | 6765 |  |  |  |  |  |
|  | TEmotion | 671 |  |  |  |  |  |
|  | Residual | 12265 |  |  |  |  |  |

Supplementary Table 2b. Expt. 2 output for the converged Error model:

glmer (Errors ~ 1 + Perceiver Sex * Target Sex * Target Race * Target Emotion + (1 + Target Emotion |Participant) + (1|item))

|  | Effect | Variance | Estimate | *SE* | *t* | *p* |
| --- | --- | --- | --- | --- | --- | --- |
| Fixed Effects | |  |  |  |  |  |
|  | (Intercept) |  | -3.14 | 0.07 | -47.32 | <.001*** |
|  | PSex |  | 0.18 | 0.11 | 1.60 | .111 |
|  | PRace |  | -0.12 | 0.11 | -1.11 | .266 |
|  | TSex |  | -0.07 | 0.08 | -0.80 | .425 |
|  | TRace |  | 0.05 | 0.08 | 0.63 | .531 |
|  | TEmotion |  | -0.14 | 0.10 | -1.35 | .176 |
|  | PSex*PRace |  | -0.20 | 0.22 | -0.91 | .361 |
|  | PSex*TSex |  | 0.06 | 0.11 | 0.49 | .621 |
|  | PRace*TSex |  | 0.04 | 0.11 | 0.39 | .694 |
|  | PSex*TRace |  | 0.18 | 0.11 | 1.58 | .115 |
|  | PRace*TRace |  | -0.10 | 0.11 | -0.91 | .366 |
|  | TSex*TRace |  | 0.15 | 0.17 | 0.88 | .378 |
|  | PSex*TEmotion |  | -0.15 | 0.14 | -1.06 | .288 |
|  | PRace*TEmotion |  | 0.10 | 0.14 | 0.71 | .476 |
|  | TSex*TEmotion |  | 0.54 | 0.17 | 3.25 | .001** |
|  | TRace*TEmotion |  | -0.23 | 0.17 | -1.37 | .171 |
|  | PSex*PRace*TSex |  | -0.11 | 0.22 | -0.49 | .626 |
|  | PSex*PRace*TRace |  | -0.11 | 0.22 | -0.50 | .617 |
|  | PSex*TSex*TRace |  | -0.26 | 0.22 | -1.17 | .243 |
|  | PRace*TSex*TRace |  | 0.20 | 0.22 | 0.88 | .377 |
|  | PSex*PRace*TEmotion |  | 0.35 | 0.29 | 1.24 | .215 |
|  | PSex*TSex*TEmotion |  | -0.83 | 0.22 | -3.70 | <.001*** |
|  | PRace*TSex*TEmotion |  | -0.12 | 0.22 | -0.55 | .584 |
|  | PSex*TRace*TEmotion |  | 0.37 | 0.22 | 1.67 | .094 |
|  | PRace*TRace*TEmotion |  | -0.77 | 0.22 | -3.44 | .001** |
|  | TSex*TRace*TEmotion |  | 0.30 | 0.33 | 0.90 | .366 |
|  | PSex*PRace*TSex*TRace |  | -0.49 | 0.45 | -1.10 | .271 |
|  | PSex*PRace*TSex*TEmotion |  | 0.14 | 0.45 | 0.32 | .747 |
|  | PSex*PRace*TRace*TEmotion |  | 0.27 | 0.45 | 0.60 | .551 |
|  | PSex*TSex*TRace*TEmotion |  | -0.52 | 0.45 | -1.16 | .248 |
|  | PRace*TSex*TRace*TEmotion |  | -0.15 | 0.45 | -0.34 | .734 |
|  | PSex*PRace*TSex*TRace*TEmotion |  | -1.48 | 0.89 | -1.66 | .096 |
| Random Effects | |  |  |  |  |  |
|  | Item (Intercept) | 0.12 |  |  |  |  |
|  | Subject (Intercept) | 0.45 |  |  |  |  |
|  | TEmotion | 0.33 |  |  |  |  |

Supplementary Table 3ai. Expt. 3a output for the converged RT model:

lmer (RT ~ 1 + Perceiver Sex * Target Sex * Target Race * Target Emotion + (1 + Target Emotion |Participants) + (1|item))

|  | Effect | Variance | Estimate | *SE* | *df* | *t* | *p* |
| --- | --- | --- | --- | --- | --- | --- | --- |
| Fixed Effects | |  |  |  |  |  |  |
|  | (Intercept) |  | 568.22 | 8.97 | 100 | 63.36 | <.001*** |
|  | PSex |  | -12.99 | 17.61 | 93 | -.74 | .463 |
|  | TSex |  | -.93 | 3.95 | 117 | -.24 | .815 |
|  | TRace |  | 7.28 | 3.95 | 117 | 1.84 | .068 |
|  | TEmotion |  | -21.91 | 4.80 | 151 | -4.57 | <.001*** |
|  | PSex*TSex |  | -4.54 | 3.99 | 10941 | -1.14 | .255 |
|  | PSex*TRace |  | -1.74 | 3.99 | 10943 | -.44 | .662 |
|  | TSex*TRace |  | 9.71 | 7.90 | 117 | 1.23 | .222 |
|  | PSex*TEmotion |  | -1.08 | 6.75 | 92 | -.16 | .873 |
|  | TSex*TEmotion |  | 12.36 | 7.90 | 117 | 1.56 | .121 |
|  | TRace*TEmotion |  | -26.37 | 7.90 | 117 | -3.34 | .001** |
|  | PSex*TSex*TRace |  | -.03 | 7.98 | 10941 | .00 | .997 |
|  | PSex*TSex*TEmotion |  | -10.36 | 7.98 | 10941 | -1.30 | .195 |
|  | PSex*TRace*TEmotion |  | 1.00 | 7.99 | 10940 | .13 | .901 |
|  | TSex*TRace*TEmotion |  | 30.86 | 15.81 | 117 | 1.95 | .053 |
|  | PSex*TSex*TRace*TEmotion |  | -7.44 | 15.97 | 10941 | -.47 | .641 |
| Random Effects | |  |  |  |  |  |  |
|  | Item (Intercept) | 372 |  |  |  |  |  |
|  | Subject (Intercept) | 7267 |  |  |  |  |  |
|  | TEmotion | 701 |  |  |  |  |  |
|  | Residual | 11187 |  |  |  |  |  |

Supplementary Table 3aii. Expt. 3a output for the converged Error model:

glmer (Errors ~ 1 + Perceiver Sex * Target Sex * Target Race * Target Emotion + (1 + Target Emotion |Participant) + (1|item))

|  | Effect | Variance | Estimate | *SE* | *t* | *p* |  |
| --- | --- | --- | --- | --- | --- | --- | --- |
| Fixed Effects | |  |  |  |  |  |  |
|  | (Intercept) |  | -3.45 | .12 | -29.45 | <.001*** |  |
|  | PSex |  | .21 | .21 | 1.00 | .316 |  |
|  | TSex |  | .06 | .12 | .49 | .626 |  |
|  | TRace |  | .15 | .12 | 1.23 | .219 |  |
|  | TEmotion |  | -.12 | .15 | -.80 | .423 |  |
|  | PSex*TSex |  | -.07 | .17 | -.39 | .699 |  |
|  | PSex*TRace |  | -.09 | .17 | -.51 | .610 |  |
|  | TSex*TRace |  | .26 | .25 | 1.08 | .281 |  |
|  | PSex*TEmotion |  | -.06 | .20 | -.31 | .755 |  |
|  | TSex*TEmotion |  | .32 | .25 | 1.31 | .190 |  |
|  | TRace*TEmotion |  | -.74 | .25 | -3.02 | .003** |  |
|  | PSex*TSex*TRace |  | .34 | .35 | .99 | .324 |  |
|  | PSex*TSex*TEmotion |  | -.72 | .35 | -2.06 | .040* |  |
|  | PSex*TRace*TEmotion |  | -.46 | .35 | -1.32 | .188 |  |
|  | TSex*TRace*TEmotion |  | 1.25 | .49 | 2.55 | .011* |  |
|  | PSex*TSex*TRace*TEmotion |  | -.11 | .70 | -.16 | .875 |  |
| Random Effects | |  |  |  |  |  |  |
|  | Item (Intercept) | .23 |  |  |  |  |  |
|  | Subject (Intercept) | .77 |  |  |  |  |  |
|  | TEmotion | .19 |  |  |  |  |  |

Supplementary Table 3bi. Expt. 3b output for the converged RT model:

lmer (RT ~ 1 + Perceiver Sex * Target Sex * Target Race * Target Emotion + (1 + Target Emotion |Participants) + (1|item))

|  | Effect | Variance | Estimate | *SE* | *df* | *t* | *p* |
| --- | --- | --- | --- | --- | --- | --- | --- |
| Fixed Effects | |  |  |  |  |  |  |
|  | (Intercept) |  | 597.36 | 9.17 | 108 | 65.15 | <.001*** |
|  | PSex |  | 20.19 | 18.03 | 101 | 1.12 | .265 |
|  | TSex |  | 5.99 | 4.04 | 115 | 1.48 | .141 |
|  | TRace |  | 6.09 | 4.04 | 115 | 1.51 | .134 |
|  | TEmotion |  | -23.94 | 4.86 | 145 | -4.93 | <.001*** |
|  | PSex*TSex |  | 4.59 | 4.51 | 11706 | 1.02 | .308 |
|  | PSex*TRace |  | -4.01 | 4.51 | 11703 | -.89 | .374 |
|  | TSex*TRace |  | -1.07 | 8.07 | 115 | -.13 | .895 |
|  | PSex*TEmotion |  | -5.92 | 7.05 | 101 | -.84 | .403 |
|  | TSex*TEmotion |  | 23.92 | 8.07 | 115 | 2.96 | .004** |
|  | TRace*TEmotion |  | -20.98 | 8.07 | 115 | -2.60 | .011* |
|  | PSex*TSex*TRace |  | -4.38 | 9.01 | 11704 | -.49 | .627 |
|  | PSex*TSex*TEmotion |  | -21.36 | 9.01 | 11701 | -2.37 | .018* |
|  | PSex*TRace*TEmotion |  | -8.70 | 9.01 | 11704 | -.97 | .335 |
|  | TSex*TRace*TEmotion |  | 9.70 | 16.14 | 115 | .60 | .549 |
|  | PSex*TSex*TRace*TEmotion |  | -2.46 | 18.02 | 11703 | -.14 | .891 |
| Random Effects | |  |  |  |  |  |  |
|  | Item (Intercept) | 359 |  |  |  |  |  |
|  | Subject (Intercept) | 8238 |  |  |  |  |  |
|  | TEmotion | 754 |  |  |  |  |  |
|  | Residual | 15215 |  |  |  |  |  |

Supplementary Table 3bii. Expt. 3b output for the converged Error model:

glmer (Errors ~ 1 + Perceiver Sex * Target Sex * Target Race * Target Emotion + (1 + Target Emotion |Participant) + (1|item))

|  | Effect | Variance | Estimate | *SE* | *t* | *p* |
| --- | --- | --- | --- | --- | --- | --- |
| Fixed Effects | |  |  |  |  |  |
|  | (Intercept) |  | -3.31 | .10 | -33.03 | <.001*** |
|  | PSex |  | .09 | .17 | .51 | .607 |
|  | TSex |  | .09 | .12 | .78 | .438 |
|  | TRace |  | .08 | .12 | .71 | .481 |
|  | TEmotion |  | -.29 | .15 | -1.93 | .054 |
|  | PSex*TSex |  | -.10 | .16 | -.58 | .559 |
|  | PSex*TRace |  | .09 | .16 | .56 | .578 |
|  | TSex*TRace |  | -.04 | .24 | -.19 | .851 |
|  | PSex*TEmotion |  | -.38 | .22 | -1.75 | .080 |
|  | TSex*TEmotion |  | .33 | .24 | 1.38 | .168 |
|  | TRace*TEmotion |  | -.48 | .24 | -2.02 | .043* |
|  | PSex*TSex*TRace |  | .77 | .33 | 2.36 | .018* |
|  | PSex*TSex*TEmotion |  | -1.28 | .33 | -3.92 | <.001*** |
|  | PSex*TRace*TEmotion |  | -.75 | .33 | -2.30 | .022* |
|  | TSex*TRace*TEmotion |  | -.26 | .47 | -.55 | .585 |
|  | PSex*TSex*TRace*TEmotion |  | .41 | .65 | .63 | .530 |
| Random Effects | |  |  |  |  |  |
|  | Item (Intercept) | .23 |  |  |  |  |
|  | Subject (Intercept) | .54 |  |  |  |  |
|  | TEmotion | .44 |  |  |  |  |

Supplementary Table 3ci. Expt. 3c output for the converged RT model:

lmer (RT ~ 1 + Perceiver Sex * Target Sex * Target Race * Target Emotion + (1 + Target Emotion |Participants) + (1|item))

|  | Effect | Variance | Estimate | *SE* | *df* | *t* | *p* |
| --- | --- | --- | --- | --- | --- | --- | --- |
| Fixed Effects | |  |  |  |  |  |  |
|  | (Intercept) |  | 536.70 | 7.55 | 105 | 71.05 | <.001*** |
|  | PSex |  | 29.48 | 14.94 | 101 | 1.97 | .051 |
|  | TSex |  | -.61 | 2.82 | 117 | -.22 | .830 |
|  | TRace |  | 4.04 | 2.82 | 117 | 1.43 | .155 |
|  | TEmotion |  | -15.97 | 3.53 | 138 | -4.53 | <.001*** |
|  | PSex*TSex |  | 5.54 | 3.51 | 11784 | 1.58 | .115 |
|  | PSex*TRace |  | 6.50 | 3.51 | 11786 | 1.85 | .065 |
|  | TSex*TRace |  | 4.65 | 5.65 | 117 | .82 | .412 |
|  | PSex*TEmotion |  | 8.89 | 5.50 | 100 | 1.62 | .109 |
|  | TSex*TEmotion |  | 16.76 | 5.65 | 117 | 2.97 | .004** |
|  | TRace*TEmotion |  | 12.15 | 5.65 | 117 | 2.15 | .034* |
|  | PSex*TSex*TRace |  | 11.83 | 7.03 | 11785 | 1.68 | .092 |
|  | PSex*TSex*TEmotion |  | -18.49 | 7.03 | 11784 | -2.63 | .009** |
|  | PSex*TRace*TEmotion |  | -.06 | 7.03 | 11785 | -.01 | .993 |
|  | TSex*TRace*TEmotion |  | 10.38 | 11.30 | 117 | .92 | .360 |
|  | PSex*TSex*TRace*TEmotion |  | .82 | 14.06 | 11787 | .06 | .953 |
| Random Effects | |  |  |  |  |  |  |
|  | Item (Intercept) | 157 |  |  |  |  |  |
|  | Subject (Intercept) | 5666 |  |  |  |  |  |
|  | TEmotion | 459 |  |  |  |  |  |
|  | Residual | 9325 |  |  |  |  |  |

Supplementary Table 3cii. Expt. 3c output for the converged Error model:

glmer (Errors ~ 1 + Perceiver Sex * Target Sex * Target Race * Target Emotion + (1 + Target Emotion |Participant) + (1|item))

|  | Effect | Variance | Estimate | *SE* | *t* | *p* |
| --- | --- | --- | --- | --- | --- | --- |
| Fixed Effects | |  |  |  |  |  |
|  | (Intercept) |  | -3.00 | .09 | -34.76 | <.001*** |
|  | PSex |  | -.01 | .15 | -.06 | .953 |
|  | TSex |  | .03 | .10 | .33 | .739 |
|  | TRace |  | .02 | .10 | .23 | .821 |
|  | TEmotion |  | .00 | .12 | -.01 | .994 |
|  | PSex*TSex |  | -.08 | .15 | -.52 | .604 |
|  | PSex*TRace |  | .13 | .15 | .88 | .380 |
|  | TSex*TRace |  | .01 | .20 | .03 | .974 |
|  | PSex*TEmotion |  | -.18 | .16 | -1.14 | .256 |
|  | TSex*TEmotion |  | .45 | .20 | 2.25 | .024* |
|  | TRace*TEmotion |  | .08 | .20 | .42 | .676 |
|  | PSex*TSex*TRace |  | .10 | .30 | .34 | .733 |
|  | PSex*TSex*TEmotion |  | -.97 | .30 | -3.26 | .001** |
|  | PSex*TRace*TEmotion |  | .61 | .30 | 2.07 | .039* |
|  | TSex*TRace*TEmotion |  | .27 | .40 | .66 | .507 |
|  | PSex*TSex*TRace*TEmotion |  | -.72 | .59 | -1.22 | .222 |
| Random Effects | |  |  |  |  |  |
|  | Item (Intercept) | .14 |  |  |  |  |
|  | Subject (Intercept) | .43 |  |  |  |  |
|  | TEmotion | .09 |  |  |  |  |
